# *Aeromonas salmonicida* activates rainbow trout IgM^+^ B cells signalling through Toll like receptors

**DOI:** 10.1038/s41598-020-73999-w

**Published:** 2020-10-08

**Authors:** Irene Soleto, Esther Morel, Estefanía Muñoz-Atienza, Patricia Díaz-Rosales, Carolina Tafalla

**Affiliations:** Fish Immunology and Pathology Laboratory, Animal Health Research Center (CISA-INIA), Valdeolmos, Madrid, Spain

**Keywords:** Immunology, Innate immunity

## Abstract

As B cells are singularly equipped with a B cell receptor (BCR) and a range of innate receptors, they are able to integrate both antigen-specific and innate signals, with the latter being essential to reach an adequate level of activation. Whether teleost B cells sense pathogens through innate mechanisms has not yet been explored, despite the fact that fish B cells display a wider array of innate receptors than many mammalian B cell subsets. Hence, in the current study, we have investigated the effects of inactivated *Aeromonas salmonicida*, a Gram negative rainbow trout pathogen, on trout splenic IgM^+^ B cells in vitro in the presence or absence of different inhibitors of Toll-like receptor (TLR) signalling, to establish to what degree innate signals are contributing to the activation of B cells in teleosts. Our results demonstrate that most of the effects that *A. salmonicida* exerts on trout IgM^+^ B cells are significantly blocked in the presence of inhibitors of MyD88 and TRIF, important nodes in TLR signal pathways. Thus, the data presented demonstrates that, also in teleost, TLR signalling is essential for the activation of IgM^+^ B cells. These results will be useful for the future optimization of novel vaccines and adjuvants.

## Introduction

The discovery of host-encoded pattern recognition receptors (PRRs) that sense common molecular patterns from infectious agents and the demonstration of their role in triggering early inflammatory processes, has been a key milestone to fully understand how the immune response is organized^1^. These receptors sense pathogen-associated molecular patterns (PAMPs) or danger-associated molecular patterns (DAMPs) and activate cells to initiate a cascade of responses that leads to the production of cytokines or antimicrobial compounds that orchestrate an inflammatory response^2^. A further consequence of PRR engagement is the activation of antigen presenting cells to initiate antigen presentation to T cells. Therefore, the ligation of PRRs benefits the host in two ways. First, it activates an innate response aimed at directly killing pathogens, while at the same time, it initiates an antigen-specific adaptive immune response that provides a second layer of protection in case it is needed^1,2^. Despite having established their evident effects on the adaptive immune response, PRR-mediated responses have been historically associated with innate immune cell populations such as dendritic cells and macrophages. Evidence gathered in the recent years, however, has pointed to a key role of B cell-intrinsic PRR activation in modelling specific antibody responses^3^.

Toll-like receptors (TLRs) constitute the most studied group of PRRs, and have been identified in all animal groups from invertebrates to human^4^. TLRs are synthetized in the endoplasmic reticulum, transported to the Golgi complex, and then delivered to the cellular membrane or to intracellular compartments like endosomes. Thus, they are type I transmembrane proteins. They are formed by an ectodomain containing several leucine-rich repeats responsible for the recognition of PAMPs; a transmembrane domain; and an intracellular Toll-interleukin 1 (IL-1) receptor (TIR) domain that mediates downstream signal transduction^5^. Once activated, the TIR domain of the TLRs recruits specific adaptor molecules which provoke the subsequent activation of the inflammatory response. Two different TLR signalling pathways have been described depending on the adaptor molecule that is recruited by TIR, a Myeloid Differentiation Primary Response protein 88 (MyD88)-dependent pathway and a TRIF-dependent pathway which is completely independent of MyD88. In mammals, MyD88 is used by all TLRs except TLR3. TLR3 signals exclusively through TRIF, while TLR4 can use both adaptors^5^. In mammals, TLRs can be further divided in two subgroups depending on their cellular location. One group is composed by TLR1, TLR2, TLR4, TLR5, TLR6, TLR10 and TLR11, expressed on the cell surface where they recognize microbial membrane components such us LPS or zymosan^5^. Another group is composed by TLR3, TLR7, TLR8 and TLR9 mainly expressed in intracellular vesicles such as endosomes or lysosomes, where they mainly recognize nucleic acids from intracellular pathogens^5^.

TLR expression on B cells varies greatly among mammalian species and different B cell subsets^6^. For example, murine naïve B cells express TLR4 and consequently, are responsive to its ligand, LPS, that stimulates their proliferation, cytokine production and IgM secretion^7^. In contrast, human naïve B cells are unresponsive to LPS, due to a lack of TLR4 expression, although TLR4 expression on B cells can be elevated in response to certain inflammatory diseases^8^. Human and murine B cells express other TLRs such as for example TLR7 and TLR9 and therefore can be activated by their ligands. Nevertheless, in general, murine B cells are more responsive to TLR ligands than human B cells, as the latter only express constitutively less accessible endosomal TLRs^6^. In contrast, human memory B cells express several TLRs at constitutively high levels^9^, regulating through this expression a polyclonal activation of the entire pool of memory B cells. Additionally, multiple recent investigations have revealed a role for this B cell-intrinsic TLR expression in B cell development^10^ and differentiation^11,12^, highlighting the great potential of TLR ligands as adjuvants.

In teleost fish, at least 20 different TLRs have been described in different species^13^. However, the PAMPs that are recognized by each of these receptors have not been established for most of these molecules, and ligand specificity has been mostly presumed based on transcriptional studies^14^. Therefore, still much work is required in these species to fully understand the functionality and regulation of each of these TLRs. Interestingly, rainbow trout B cells were shown to actively transcribe all TLR sequences known to date in this species^15^. Thus, how B cells respond to different TLR ligands has been a matter of recent interest by many fish immunologists^16–18^. Despite these recent studies, the precise contribution of innate signalling to B cell activation by pathogens or vaccines had not yet been addressed. Thus, in the current study, we have studied the effect of inactivated *Aeromonas salmonicida* on trout splenic IgM^+^ B cells in vitro in the presence or absence of different inhibitors of TLR signalling, to establish to what degree innate signals are contributing to the activation of B cells in teleost. *A. salmonicida* is a Gram negative bacteria and the cause of furunculosis, one of the most important fish health problems in salmonid aquaculture^19^. Although commercial vaccines are able to induce long-term protection, furunculosis outbreaks are still frequent in several fresh and marine aquacultured species. Thus, our results, which provide novel information regarding the mechanisms through which fish B cells recognize bacteria and become activated, will surely be valuable for the future optimization of novel prevention strategies against this and other pathogenic bacteria.

## Results

### *Aeromonas salmonicida* is phagocytized by IgM^+^B cells

**Figure 1:**
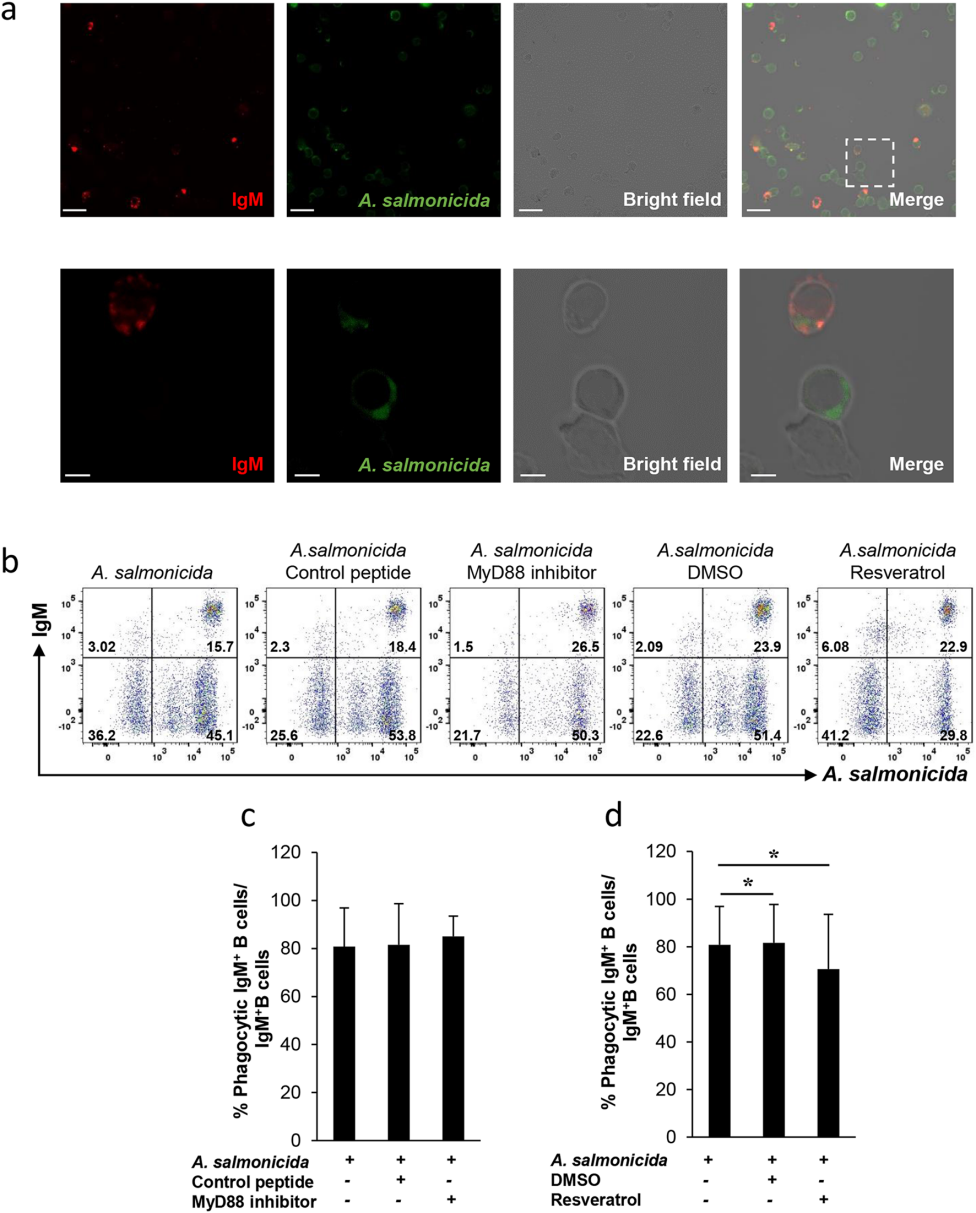
Trout B cell phagocytosis of *A. salmonicida*. Splenic leukocytes were incubated with *A. salmonicida* previously labelled with Syto BC Green at a 1:2 cell:bacteria ratio. **(a)** After 3 h, cells were stained with anti-IgM (shown as red) and plated onto poly-L-lysine coated glass slides. Samples were then analysed by confocal fluorescence microscopy. Representative confocal microscopy images include a large field (top images) and a higher magnification (lower images) showing both an IgM^+^ B cell and an IgM^-^ cell phagocyting *A. salmonicida* (scale bars, 10 µm on the large fields and 2 µm on the higher magnifications). Splenic leukocytes were incubated with MyD88 inhibitor peptide (100 µM), the control peptide (100 µM), resveratrol (50 µM), the same volume of DMSO or media alone for 1 h. Thereafter, splenocytes were incubated with *A. salmonicida* labelled with Syto BC Green. Controls without bacteria were also included. After 3 h, cells were stained with anti-trout IgM-APC and analysed by flow cytometry. Representative dot plot from one individual fish is shown (**b**), along with the quantification of the percentage of phagocytic IgM^+^ B cells (cells in the upper right quadrant) among total IgM^+^ cells (cells in upper quadrants) after each treatment (mean + SD; n = 7 individual fish) (**c, d**). Asterisks denote significant differences between groups as indicated (*P ≤ 0.05).

Prior to characterizing the effects of *A. salmonicida* rainbow trout B cells, we studied whether this fish pathogen could be phagocytized by IgM^+^ B cells. For this, we labelled inactivated *A. salmonicida* with Syto BC Green and incubated splenocytes with the labelled bacteria for 3 h. Thereafter, cells were labelled with a specific anti-IgM monoclonal antibody and analysed by confocal microscopy or flow cytometry. Our results show that *A. salmonicida* can be phagocytized by IgM^+^ B cells (Fig. 1A,B), as well as by other non-IgM leukocytes (Fig. 1A,B).

Our next step then was to establish if the TLR inhibitors used throughout this work had a negative impact on the capacity of rainbow trout IgM^+^ B cells to phagocytize *A. salmonicida*. For this, we pre-incubated splenocytes for 1 h with the MyD88 inhibitor peptide or resveratrol. As negative controls, we used a control peptide or DMSO which is the solvent in which resveratrol is dissolved. After this time, we incubated the cells with labelled inactivated *A. salmonicida* for 3 h and analysed the phagocytic capacity by flow cytometry (Fig. 1B). The MyD88 inhibitor peptide did not have a negative effect on the capacity of IgM^+^ B cells to internalize *A. salmonicida* (Fig. 1C), while resveratrol significantly inhibited the internalization (Fig. 1D). Similarly, resveratrol has been shown to reduce the phagocytic activity of human macrophages by down-regulating the expression of phagocytic receptors and NF-κB activity ^20^.

### *A. salmonicida* increases IgM^+^ B cell survival and has lymphoproliferative effects through a TLR-dependent mechanism

**Figure 2:**
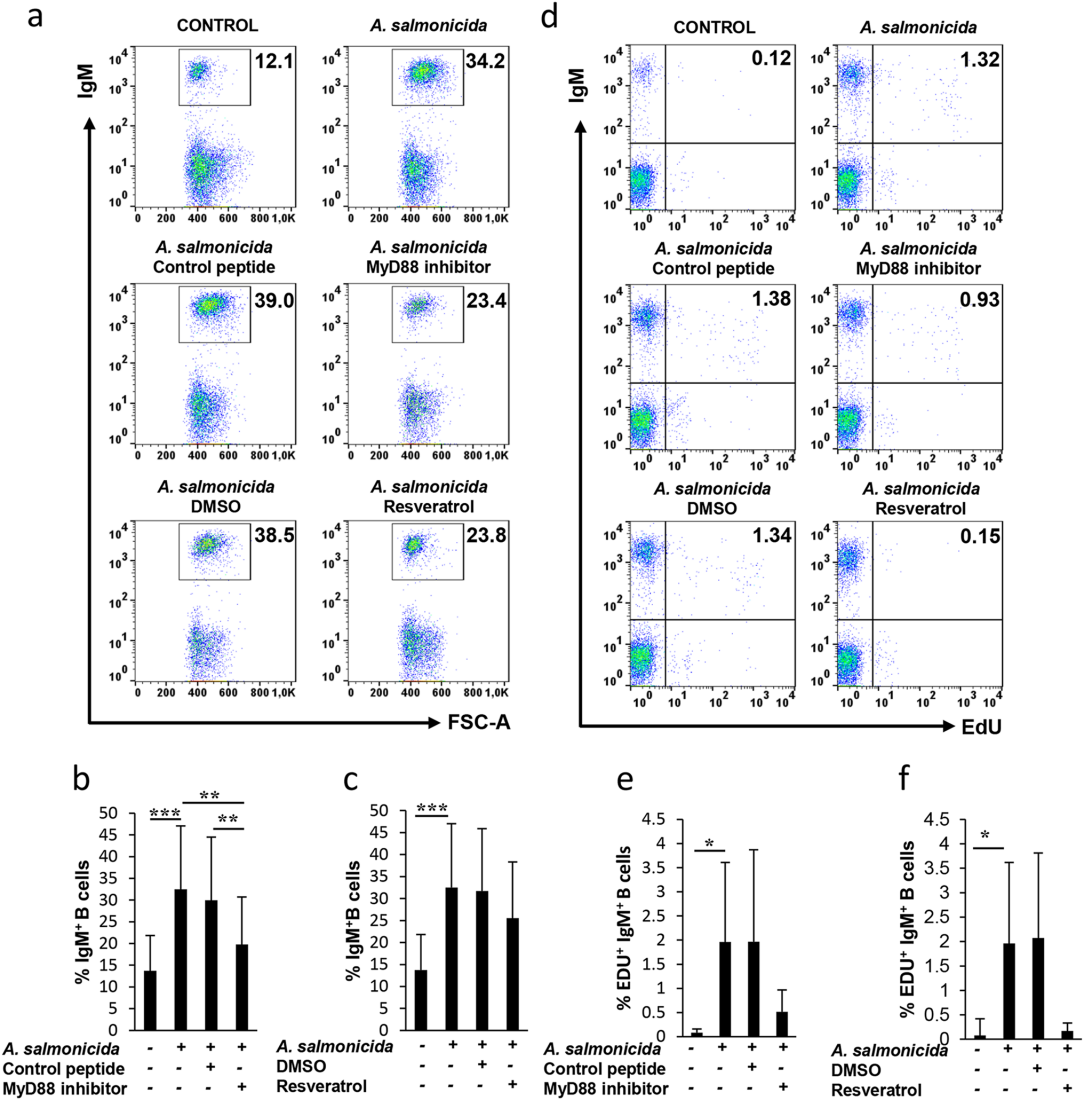
*A. salmonicida* increases IgM^+^ B cell survival and has lymphoproliferative effects. Splenocytes were incubated with the different inhibitors or left unstimulated as described in the legend of Fig. 1. After 1 h, cells were stimulated with *A. salmonicida* at a ratio 1:2 (cell:bacteria). Controls without bacteria were also included. After 3 days of incubation at 20 ºC, cells were labelled with anti-trout IgM-APC and the percentage of IgM^+^ B cells in the cultures determined by flow cytometry. Representative dot plots are shown **(a)**, together with a quantification of the percentage of IgM^+^ B cells in cultures (mean + SD; n = 12) **(b,c)**. The lymphoproliferative effects of *A. salmonicida* on B cells were determined in parallel. For this, cells were pre-treated with the inhibitors and then stimulated with the bacteria as described above. After 3 days of incubation, splenic leukocytes were incubated with EdU for an additional 24 h. At that point, cells were labelled with anti-trout IgM-APC and the percentage of proliferating cells determined as described in the Materials and Methods section. Representative dot plots are shown **(d)**, together with a quantification of the percentage of proliferating IgM^+^ B cells (mean + SD; n = 9) **(e,f)**. Asterisks denote significant differences between groups as indicated (*P ≤ 0.05; ***P ≤ 0.005).

Next, we investigated the effects of *A. salmonicida* on the survival of rainbow trout IgM^+^ B cells. To this end, splenocytes were exposed to the different TLR inhibitors or their respective controls for 1 h and then incubated with the bacteria for 3 days. Controls without bacteria were also included. After this time, cells were labelled with anti-IgM and DAPI (to determine cell viability) and analysed following the gating strategy described in Supplementary Fig S1, after establishing that none of the treatments had a significant impact on cell viability (Fig. S1). Resveratrol provoked a moderate but non-significant decrease in the number of cells within the lymphoid gate, however the percentage of live cells within the gated population was never affected (Fig. S1). In these conditions, we established that *A. salmonicida* significantly increased the percentage of IgM^+^ B cells in the cultures, effect that was reverted by the MyD88 inhibitor peptide but not by its respective control or by resveratrol (Fig. 2A–C). Similar results were obtained when the absolute number of total IgM^+^ B cells was determined (Fig. S2). To establish whether this increased percentage of B cells was due to a lymphoproliferative effect of *A. salmonicida,* a proliferation assay was carried out in parallel. As a first step, we established that none of the inhibitors or the controls used in the experiments were able to induce proliferation in the absence of *A. salmonicida* (Fig. S3). *A. salmonicida*, however, induced a low but significant proliferation of IgM^+^ B cells (Fig. 2D–F). Although these mild proliferative effects were down-regulated in the presence of the MyD88 inhibitor peptide and resveratrol, the differences compared to the values reached by *A. salmonicida* alone were not significant. These results demonstrate that *A. salmonicida* is acting mainly as a survival factor for rainbow trout IgM^+^ B cells, through a mechanism that involves MyD88.

### *A. salmonicida* increases MHC II surface expression in IgM^+^ B cells through a TLR-dependent mechanism

**Figure 3:**
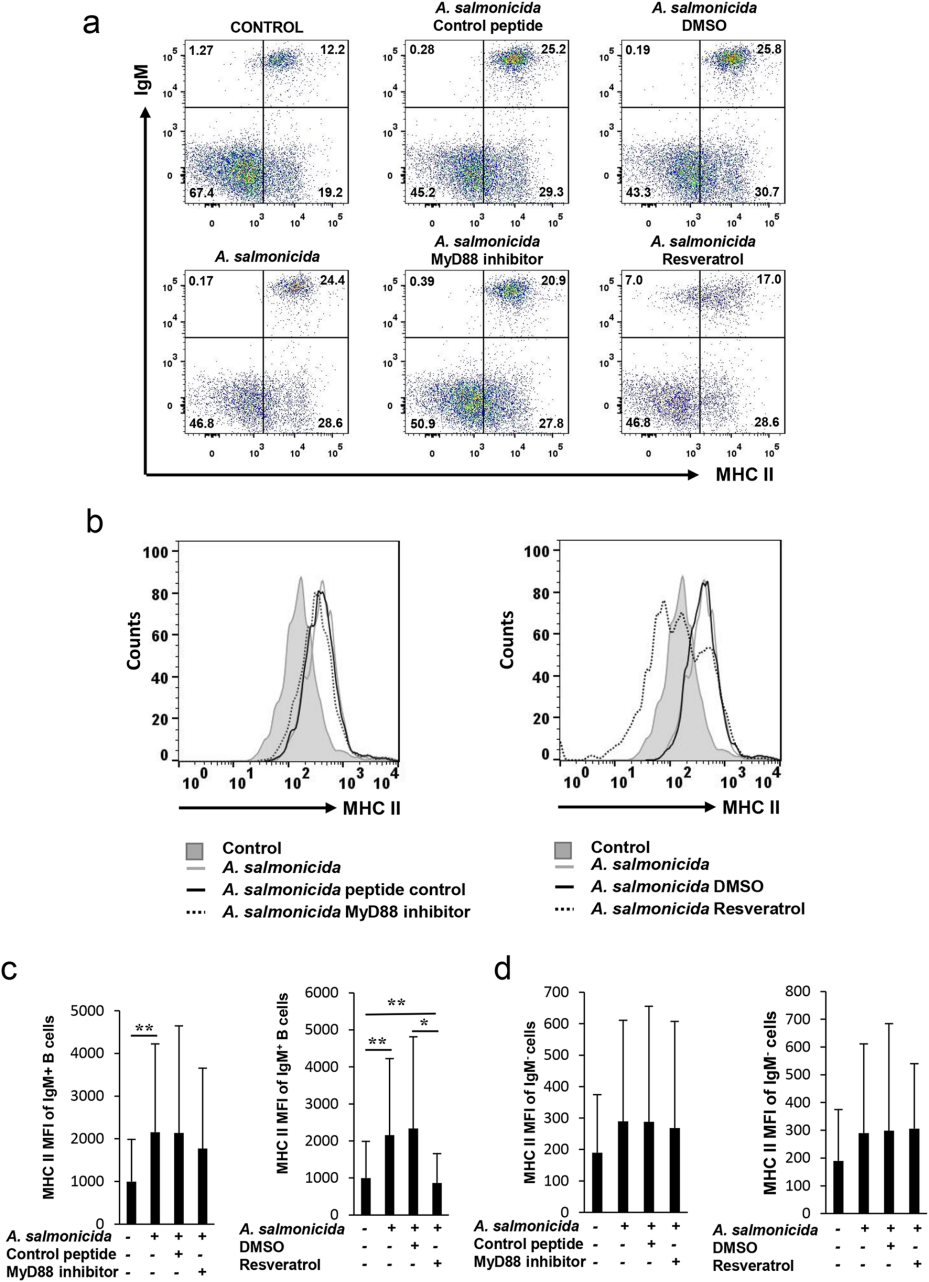
*A. salmonicida* increases the expression of surface MHC II on IgM^+^ B cells. Splenocytes were incubated with the different inhibitors or left unstimulated as described in the legend of Fig. 1. After 1 h, cells were stimulated with *A. salmonicida* at a ratio 1:2 (cell:bacteria). Controls without bacteria were also included. After 3 days of incubation at 20ºC, cells were labelled with anti-trout IgM-FITC and anti-trout MHC II-APC and analysed by flow cytometry. Representative dot plots **(a)** and histograms **(b)** showing MHC II expression levels in IgM^+^ B cells from one representative fish are included, along with the quantification of MHC II mean fluorescence intensity (MFI) values in IgM^+^ B cells **(c)** and IgM- cells **(d)** (mean + SD; n = 12). Asterisks denote significant differences between groups as indicated (*P ≤ 0.05; **P ≤ 0.01).

IgM^+^ B cells as professional antigen presenting cells express MHC II on their cell surface^21^. Thus, we decided to study if upon an encounter with *A. salmonicida* IgM^+^ B cells modified the expression of MHC II on the surface and whether this effect was regulated by TLR signalling. For this, again we incubated splenocytes for 1 h with the inhibitors or their respective controls, and then stimulated the cells with inactivated *A. salmonicida*. After 72 h of incubation at 20 ºC, the cells were labelled with anti- IgM and anti-MHC II monoclonal antibodies, analysed by multicolor flow cytometry and the fluorescence intensity of MHC II measured. A clear effect of *A. salmonicida* on the levels of surface MHC II on IgM^+^ B cells was visible (Fig. 3A–C), effect that was reverted by resveratrol (Fig. 3A–C). In this case, the MyD88 inhibitor peptide was not able of reverting the increase in the levels of surface MHC II provoked by the bacteria (Fig. 3A–C). Interestingly, in these cultures, the levels of surface MHC II were not significantly increased by the bacteria in IgM^-^ cells (Fig. 3D), suggesting a preferential role of IgM^+^ B cells among splenocytes in the presentation of *A. salmonicida*.

### *A. salmonicida* increases the number of antibody secreting cells in splenocyte cultures through a TLR-dependent mechanism

**Figure 4:**
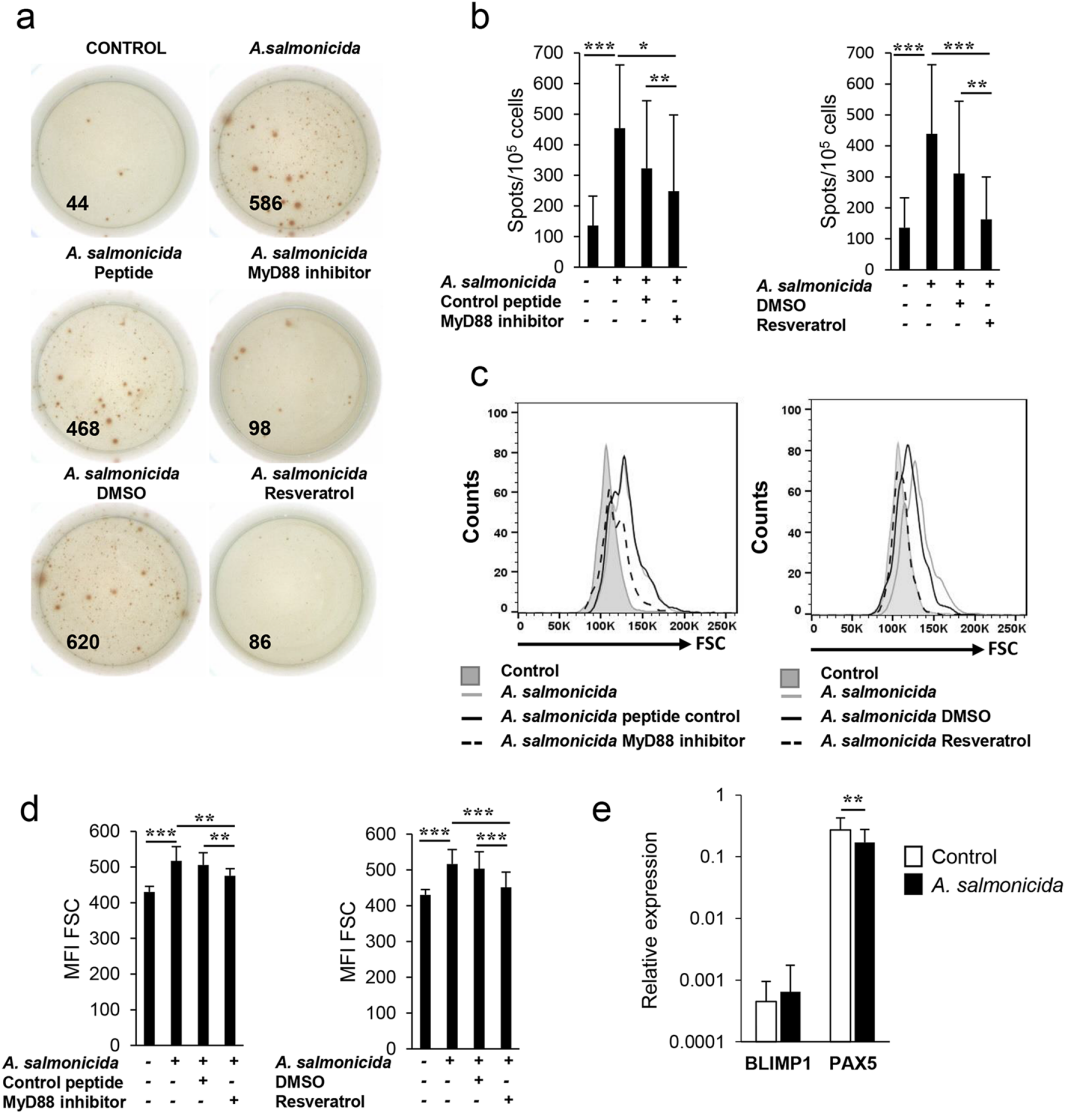
*A. salmonicida* differentiates B cells to IgM-secreting plasmablasts. Splenocytes were incubated with inhibitors or left unstimulated as described in the legend of Fig. 1. After 1 h, cells were stimulated with *A. salmonicida* at a ratio 1:2 (cell:bacteria). Controls without bacteria were also included. After 48 h at 20 ºC, cells were plated into ELISPOT plates previously coated with anti-trout IgM and incubated for a further 24 h. At this point, cells were washed and a biotinylated anti-trout IgM used to detect number of spot forming cells. Images from a representative fish are shown **(a)** together with a quantification of the number of IgM-secreting cells (mean + SD; n = 12) **(b).** Splenocytes pre-treated with the inhibitors, and stimulated with *A. salmonicida* were also analysed by flow cytometry after 72 h of incubation with the bacteria. IgM^+^ B cells were gated and the MFI of their forward scatter (FSC), indicative of cell size, determined. Representative histograms are shown (**c**) along with a quantification of FSC MFI values in IgM^+^ B cells (mean + SD; n = 12) (**d**). In another experiment, splenocyte cultures were stimulated with *A. salmonicida* or left untreated. After 24 h, IgM^+^ B cells were isolated by flow cytometry, RNA extracted and the levels of transcription of Blimp-1 and Pax5 determined by real time PCR as described in the Materials and Methods section. Expression relative to the endogenous control EF-1α was calculated for each sample, and is shown as mean + SD (n = 7) (**e**). Asterisks denote significant differences between groups as indicated (*P ≤ 0.05; **P ≤ 0.01; ***P ≤ 0.005).

Next, to establish if *A. salmonicida* can induce on its own the differentiation of IgM^+^ B cells to IgM-secreting plasmablasts/plasma cells, we determined the effect that *A. salmonicida* had in the number of IgM-secreting cells through an ELISPOT assay. *A. salmonicida* significantly increased the number or IgM secreting cells in splenocytes cultures after 3 days of treatment (Fig. 4A), effect that was significantly reversed in the presence of the MyD88 inhibitor peptide or resveratrol, but not by their respective controls (Fig. 4A,B). Because a differentiation of naïve B cells to plasma-like cells usually implies an increase in size, we also determined by flow cytometry the impact of *A. salmonicida* on the size (forward side scatter, FSC) of splenic IgM^+^ B cells. Our results confirmed that there was a significant size increase in response to the bacteria, effect that significantly reverted in the presence of the TLR inhibitors but not in response to their controls (Fig. 4C,D). In mammals, the B cell differentiation process implies a down-regulation of the Pax5 transcription factor, as a consequence of the increased production of Blimp1^22^. In fish, whether a similar differentiation program takes place is still partially unknown and increases in IgM secretion dependent^16^ or independent^16,23,24^ of Blimp1 transcription have been reported in rainbow trout. To establish whether *A. salmonicida* induced changes in the transcription of these factors, IgM^+^ B cells exposed or not to the bacteria were isolated by flow cytometry (Fig. S4), RNA extracted and the levels of transcription of Blimp-1 and Pax5 determined by real time PCR. In our experiments, *A. salmonicida* significantly down-regulated Pax5 transcription in IgM^+^ B cells but had no effect on Blimp1 mRNA levels (Fig. 4E).

### *A. salmonicida* LPS has lower lymphoproliferative capacities than the inactivated bacteria but stronger effects on MHC II expression

**Figure 5:**
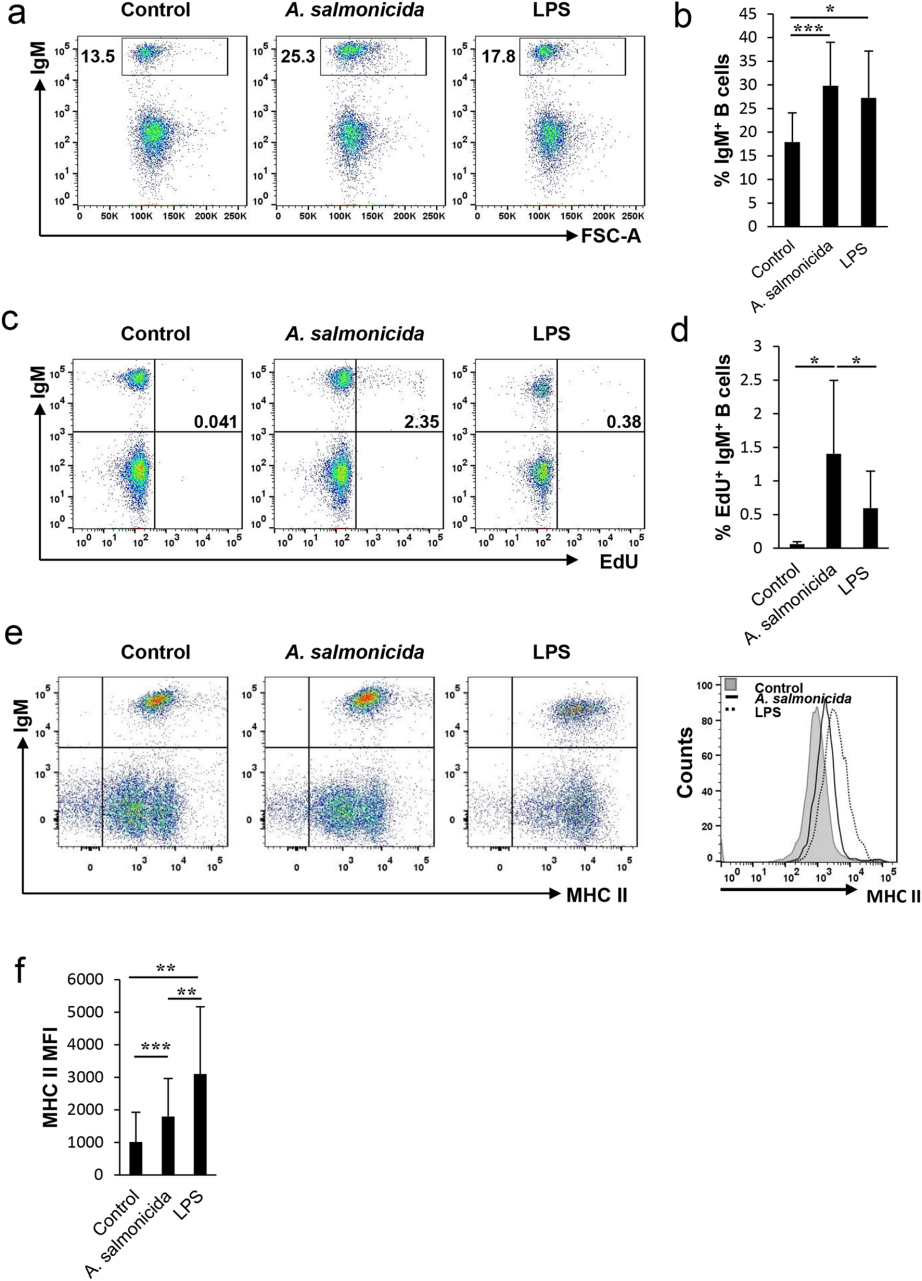
Comparative effects of *A. salmonicida* and its LPS. Splenocytes were incubated with *A. salmonicida* or an amount of LPS that corresponded to that same number of bacterial cells for 3 days at 20 ºC. Thereafter, cells were labelled anti-trout IgM-APC and analysed by flow cytometry to estimate the percentage of IgM^+^ B cells. (**a**) Representative dot plots are shown together with a quantification of average IgM^+^ B cells in cultures (mean + SD; n = 12) (**b**). At the same time the lymphoproliferative effect was measured after incubating the splenocytes with EdU for a further 24 h. At that point, cells were labelled with anti-trout IgM-APC and number of proliferating cells determined as described in Materials and Methods. Representative dot plots are shown (**c**) together with a quantification of proliferative IgM^+^ B cells (mean + SD; n = 9) (**d**). The expression of MHC II on the surface of IgM^+^ B cells was also measured after the incubation with *A. salmonicida* or its LPS for 3 days. Representative plots and histogram are shown (**e**) together with a quantification of the mean intensity fluorescence of MHC II on the surface of IgM^+^ B cells (mean + SD; n = 12) (**f**). Asterisks denote significant differences between groups as indicated (*P ≤ 0.05; **P ≤ 0.01; ***P ≤ 0.005).

Having established that *A. salmonicida* exerts positive effects on the survival, IgM secretion and MHC II expression of rainbow trout IgM^+^ B cells through a TLR-dependent mechanism, we wanted to determine whether the *A. salmonicida* LPS on its own was capable of exerting similar effects or whether the complete bacterial cell was required. For this, we isolated LPS from *A. salmonicida* and incubated splenocytes for 3 days with either the inactivated bacteria or an amount of LPS that corresponded to that same amount of bacterial cells. Both the inactivated bacteria and the LPS were capable of significantly increasing the percentage of IgM^+^ B cells in splenocyte cultures after 3 days of stimulation (Fig. 5A,B). Regarding the lymphoproliferative effects, only the inactivated bacteria induced a significant proliferation of IgM^+^ B cells while LPS did not (Fig. 5C,D). On the contrary, when we analysed the effects on the MHC II surface expression of IgM^+^ B cells, although both stimuli significantly increased the expression levels, the effects provoked by LPS were significantly higher than those exerted by the bacteria (Fig. 5E,F).

## Discussion

B cells are singularly equipped with a B cell receptor (BCR) along with a specific range of innate receptors. Thus, B cells are uniquely suited to integrate at the same time antigen-specific and innate signals. Similar to what occurs in innate B cells, PRR activation results in the up-regulation of activation markers and the secretion of pro-inflammatory cytokines by B cells. In addition, TLR signalling in mammalian B cells has also been shown to affect proliferation/differentiation and Ig secretion; B cell linage determination; negative selection and autoimmune responses^3,6^. However, how different TLR ligands affect different B cell subsets is quite variable among species, and dependent on the array of TLRs expressed. In general, it should be noted that, in mammals, innate B cell populations such as B1 or marginal zone (MZ) B cells respond significantly stronger than follicular B cells to stimulation with many TLR agonists^25^. In rainbow trout, naïve IgM^+^ B cells transcribe a broad range of TLRs^15^, being in consequence highly responsive to TLR agonists such as *Escherichia coli* LPS^16^ or CpGs^18^. Furthermore, rainbow trout B cells were recently shown to retain different phenotypical and functional characteristics of mammalian B1 cells such as low IgD and high IgM surface expression, extended survival in cell culture and lack of proliferation upon BCR engagement^26^ as well as a strong phagocytic capacity^27^. In this context, we thought of great interest to establish to what degree TLR signalling was contributing to the activation of B cells in this species. For this, we used *A. salmonicida*, an important rainbow trout pathogen that continues to cause mass mortalities in salmonid aquaculture worldwide. To rule out any possible bacteria-mediated effects, *A. salmonicida* was previously inactivated.

We first established whether the MyD88 and TRIF inhibitors we used throughout the study affected somehow the capacity of rainbow trout IgM^+^ B cells to phagocytize *A. salmonicida*. Interestingly, although resveratrol significantly inhibited this activity as previously reported in human macrophages^20^, the MyD88 inhibitor had no effect on the capacity of IgM^+^ B cells to internalize *A. salmonicida*. This implies that any changes in the response to the bacteria observed in the presence of the MyD88 inhibitor are strictly due to an interference with MyD88 signalling and cannot be attributed to a reduced internalization. In the case of resveratrol, however, it is not possible to rule out that the reduced activation of B cells in the presence of this inhibitor is at least in part due to the fact that the bacteria is not internalized as efficiently. Furthermore, it must be noted that resveratrol possesses a wide range of biological properties and can interfere with intracellular signalling during the inflammatory responses at different steps^20^. This might explain why most of the reversions of *A. salmonicida*-induced activation of B cells were much stronger in the case of resveratrol than in response to the MyD88 inhibitor. In fact, it should be noted that the precise effects of the two inhibitors used in this study in downstream TLR signalling has never been established in teleost fish due to a lack of specific reagents. In this context, although the work presented in this paper represents sufficient evidence of how both inhibitors are capable of blocking inflammatory responses also in fish, it might be possible that these inhibitors are not 100% efficacious in fish or that they have effects slightly different to those reported for mammalian cells*.*

Our results demonstrated that *A. salmonicida* was capable of increasing the survival of IgM^+^ B cells in splenocyte cultures. This increase seemed to be at least partially due to a lymphoproliferative effect of the bacteria on IgM^+^ B cells, however, the proliferation rates were not very high, suggesting that *A. salmonicida* is acting mainly as a survival factor for rainbow trout IgM^+^ B cells. In consequence, only the increased IgM^+^ B cell survival observed in response to the bacteria, was significantly reverted by the MyD88 inhibitor, demonstrating that the BCR alone does not account for this effect. The increase in B cell survival in response to the bacteria could be mediated by the recognition of *A. salmonicida* LPS, as similar B cell survival rates were obtained in response to the complete bacteria or its LPS. However, the complete bacteria seems to be a more suitable antigen for the induction of proliferation, as significantly lower responses were observed in response to LPS. Interestingly, previous investigations point to salmonids lacking TLR4, the main receptor responsible for LPS sensing in mammals^14^, thus, the receptor that is mediating these effects in B cells is still unknown and is an issue that should be addressed in future studies.

*A. salmonicida* also increased MHC II expression on the surface of IgM^+^ B cells, similarly to what had been previously reported in response to *E. coli* LPS^16^ or CpGs^18^. In this case, however, only resveratrol and not the MyD88 inhibitor, was capable of significantly reverting this effect. Whether this is a consequence of surface MHC II expression up-regulation requiring the internalization of the bacteria or because this effect is mediated through a TRIF-dependent mechanism is still undetermined and deserves further investigation. Remarkably, we found that the bacterial LPS on its own was capable of inducing higher levels of surface MHC II expression in IgM^+^ B cells than those observed in response to the intact bacteria. Hence, these results suggests that an internalization of the bacteria is not required to induce surface MHC II expression.

Our results also demonstrate a differentiation of rainbow trout naïve IgM^+^ B cells towards IgM-secreting cells in response to inactivated *A. salmonicida*. The increased number of IgM-secreting cells in the splenocyte cultures treated with the bacteria could be a result of an increased survival of already-existing IgM-secreting cells in the cultures as previously established in peritoneal B cells treated with BAFF^28^. However, the fact that *A. salmonicida* also increased the size of B cells and down-regulated their Pax5 mRNA levels suggested in fact a differentiation of naïve cells towards an IgM-secreting cell. All these effects were significantly reverted in the presence of both TLR inhibitors. In mammals, the contribution of B cell-intrinsic TLRs to antibody secretion has been widely documented. For example, Pasare and Medzhitov transferred purified B cells from wild type, MyD88 knockout, TLR4 knockout and CD40 knockout mice into mice that lacked mature B cells^29^. In these experiments, mice that received wild-type B cells were able to generate antigen-specific IgM and IgG responses to a combination of human serum albumin and LPS, whereas antibody production was significantly impaired in mice that received TLR4 knockout or MyD88 knockout B cells. Interestingly, mice that received CD40 knockout B cells had normal IgM titres but impaired IgG production^29^. Completely different results were obtained in another study in which TLR-deficient mice were immunized with a T cell dependent antigen (TNP-KLH) in combination with different adjuvants^30^. In this case, similar antibody responses were obtained in TLR-deficient mice when compared to wild type mice. These contradictory results led other authors to speculate that the degree of TLR help needed is highly dependent on the antigen, being TLR engagement required to a higher degree when BCR stimulation or T-cell help is limiting^31^. Given the scatter distribution of B and T cells in teleost immune tissues, lacking organized lymphoid structures such as cognate germinal centers^32^, and knowing that BCR signalling provokes less effects in fish B cells than in mammalian conventional B cells^26^, it seems plausible to hypothesize that TLR engagement plays a quite prominent role in teleost B cells. This hypothesis seems confirmed by the fact that the degree of reversion exerted by the TLR inhibitors on the effects provoked by *A. salmonicida* on trout IgM^+^ B cells was quite high, completely reverting many of them.

In conclusion, we have demonstrated that upon recognition of inactivated *A. salmonicida*, rainbow trout IgM^+^ B cells increase in number, proliferate, increase surface MHC II expression and differentiate towards IgM-secreting cells. Among these effects, the MyD88 inhibitor significantly reverted the increased IgM^+^ B cell survival and the up-regulated IgM secretion, whereas resveratrol significantly reverted the higher surface MHC II levels and the increased IgM secretion. These results highlight a large contribution of TLR signalling in the activation of B cells by the bacteria. Finally, we have also established that *A. salmonicida* LPS by itself has stronger effects on MHC II surface expression than the complete bacteria, while having lower lymphoproliferative effects. Understanding how fish B cells sense antigens is essential for the development of effective vaccines and adjuvants, therefore our work will hopefully contribute to the design of a more effective *A. salmonicida* vaccine in the near future.

## Materials and methods

### Experimental fish

Healthy specimens of rainbow trout (*Oncorhynchus mykiss*) of approximately 50–70 g were obtained from *Centro de Acuicultura El Molino* (Madrid, Spain). As previously described^18,24^, fish were maintained at the Animal Health Research Centre (CISA-INIA) laboratory at 16 ºC with a re-circulating water system and 12:12 h light:dark photoperiod. Fish were fed twice a day with a commercial diet (Skretting, Spain). Prior to any experimental procedure, fish were acclimatized to laboratory conditions for 2 weeks and during this period no clinical signs were ever observed. The experiments described comply with the Guidelines of the European Union Council (2010/63/EU) for the use of laboratory animals and were previously approved by the Ethics committee from the *Instituto Nacional de Investigación y Tecnología Agraria y Alimentaria* (INIA; Code CEEA PROEX002/17).

### Spleen leukocyte isolation

Rainbow trout were killed by benzocaine (Sigma-Aldrich) overdose and the spleen collected. Single cell suspensions were obtained as previously reported^18,24^ using 100 µm nylon cell strainers (BD Biosciences) and Leibovitz medium (L-15, Invitrogen) supplemented with 100 I.U./ml penicillin and 100 µg/ml streptomycin (P/S, Life Technologies), 10 units/ml heparin (Sigma) and 5% foetal calf serum (FCS, Life Technologies). Cell suspensions were placed onto 30/51% discontinuous Percoll (GE Healthcare) density gradients and centrifuged at 500 × *g* for 30 min at 4 ºC. The interface cells were washed twice in L-15 with 2% FCS and cells were resuspended in L-15 with 5% FCS. The viable cell concentration was determined by Trypan blue (Sigma-Aldrich) exclusion, adjusting the concentration to 2 × 10^6^ cells/ml.

### Bacteria and LPS

The Gram-negative fish pathogen *A. salmonicida* subsp. *salmonicida* CECT4237 was aerobically grown in Tryptone Soya Broth (Oxoid) at 25 ˚C. To stimulate the rainbow trout splenocytes, *A. salmonicida* grown in broth overnight to exponential phase was heat-inactivated at 65 °C for 1 h. In some experiments, LPS obtained from *A. salmonicida* was used to stimulate the cells. For this, LPS was isolated using a commercial extraction kit (iNtRON Biotechnology) following the manufacturer’s protocol. The absence of DNA and protein contamination was confirmed by SDS-PAGE and agarose electrophoresis.

### Reagents

A MyD88 inhibitor peptide was purchased from Novusbio and used at a concentration of 100 µM. A control peptide was used as a control at the same concentration. Resveratrol, obtained from Sigma, was diluted in DMSO and was used in cells at a final concentration of 50 µM. In this case, the same volume of DMSO was added to cell cultures as a negative control.

### Flow cytometry

IgM^+^ B cells populations were identified following a method previously described^18,24^. For this, leukocytes were incubated for 30 min with anti-trout IgM [mAb mouse IgG_1_ coupled to fluorescein (FITC) or allophycocyanin (APC) at 0.5 μg/ml] and anti-trout MHC II [mAb mouse IgG_1_ coupled to APC at 2 μg/ml] antibodies in staining buffer (phenol red-free L-15 media supplemented with P/S and 2% FCS). Both antibodies have been previously characterised^33,34^. After the incubation, cells were washed twice with staining buffer and analysed on a FACS Celesta flow cytometer (BD Biosciences) equipped with FACS DIVA software. In all cases, isotype controls for mouse mAbs (BD Biosciences) were tested in parallel to discard unspecific binding. Cells were stained with 4′,6-diamidino-2-phenylindole (DAPI; 0.2 μg/ml) to check cell viability and doublets were excluded following the gating strategy described in Supplementary Fig. S1. Flow cytometry analysis was performed with FlowJo 10 (TreeStar).

### Phagocytic activity

Spleen leukocytes seeded in 96-well plates (Nunc) at a cell density of 4 × 10^5^ cells/well were incubated with the MyD88 inhibitor peptide (10 µM), with resveratrol (50 µM) or with their respective controls at 20 °C. After 1 h, splenocytes were exposed to inactivated *A. salmonicida* previously labelled with 1 µM Syto BC Green (Thermo Fischer Scientific) at a cell: bacteria ratio of 1:2 or without bacteria in the case of negative controls and the phagocytosis assay was conducted following a method previously described^18^. After 3 h of incubation at 20 ºC, cells were harvested by gently pipetting and non-ingested beads removed by centrifugation (100 × *g* for 10 min at 4 ºC) over a cushion of 3% (weight/volume) BSA (Fraction V; Fisher Scientific) in PBS supplemented with 4.5% (weight/volume) D-glucose (Sigma). Cells were then resuspended in staining buffer, labelled with anti-trout IgM-APC (0.5 µg/ml) and analysed on a FACS Celesta flow cytometer following the gating strategy previously described (Fig. S1). For confocal analysis, cells labelled with anti-trout IgM-APC (5 µg/ml) were washed with serum-free L-15 medium, seeded on poly-L-lysine coated slides, and incubated at 20 °C for 30 min. Laser scanning confocal microscopy images (0.3 μm thickness) were acquired with an inverted Zeiss Axiovert LSM 880 microscope. Images were analysed with Zen 2.0 (Carl Zeiss) and Fiji (NIH) software packages.

### B cell proliferation

The Click-IT EdU Alexa Fluor 488 Flow Cytometry Assay Kit (Life technologies) was used to measure the proliferation of IgM^+^ B cells as described before^18,24^. For this, splenocytes at a concentration of 2 × 10^6^ cells per ml were incubated for 1 h with the different TLR inhibitors, their controls or media alone as described above. The cells were then stimulated with inactivated *A. salmonicida* for 3 days at 20 ºC as described above or left unstimulated. After this time, EdU (1 µM) was added to the cultures and the cells were incubated for an additional 24 h. At this point, cells were collected and stained with anti-trout IgM-APC (0.5 μg/ml). To analyse the incorporation of EdU, cells were then fixed and permeabilised with Cytofix/Cytoperm buffer for 15 min at room temperature (RT). Finally, the incorporation of EdU to the DNA was detected following the manufacturer´s instructions and then analysed by flow cytometry on a FACS Celesta flow cytometer.

### ELISPOT analysis

ELISPOT was used to quantify the number of IgM-secreting B cells as previously reported^18,24^. Splenocytes (2 × 10^6^ cells/ml) were incubated with the different TLR inhibitors, their controls or media alone for 1 h. At this point, cells were stimulated with inactivated *A. salmonicida* as described above for 48 h at 20 ºC. Cells (5 × 10^4^ cells per well) were then transferred to ELISPOT plates pre-coated with anti-trout IgM (2 µg/ml). After 24 h of incubation at 20ºC, cells were washed away 5 times with PBS and plates were blocked with 2% BSA in PBS for 1 h at RT. After blocking, biotinylated anti-trout IgM was added to the plates and incubated at 1 µg/ml for 1 h at RT. Following additional washing steps (5 times in PBS) the plates were developed using streptavidin-HRP at 100 ng/ml (Thermo Fischer Scientific) at RT for 1 h, washed again with PBS and incubated with 3-amino 9-ethylcarbazole (Sigma-Aldrich) for 30 min at RT in the dark. Substrate reaction was stopped by washing the plates with tap water. Once the membranes were dried, the number of spots in each well was determined using an AID iSpot Reader System (Autoimmun Diagnostika GmbH).

### Transcriptional analysis of isolated IgM^+^ B cells

IgM^+^ B cells populations were isolated by flow cytometry in a BD FACSAria III cell sorter (BD Biosciences) after staining spleen leukocytes with anti-trout IgM-APC as described above, using their FSC/SSC and fluorescence characteristics. In this case 7-AAD (BD Biosciences) at 2.5 µg/ml was used to check the cell viability.

Total cellular RNA was isolated from cell populations using the Power SYBR Green Cells-to-Ct Kit (Invitrogen) following manufacturer´s instructions and as described before^18,24^. RNA was treated with DNase during the process to remove genomic DNA that might interfere with the PCR reactions. Reverse transcription was also performed using the Power SYBR Green Cells-to-Ct Kit (Invitrogen) following manufacturer’s instructions. To evaluate the levels of transcription of the different genes, real time PCR was performed with a LightCycler 96 System instrument (Roche) using SYBR Green PCR core Reagents (Applied Biosystems) and specific primers previously described^24^. Each sample was measured in duplicate under the following conditions: 10 min at 95 ºC, followed by 40 amplification cycles (15 s at 95 ºC and 1 min at 60 ºC). A melting curve for each primer set was obtained by reading fluorescence every degree between 60 ºC and 95 ºC to ensure only a single product had been amplified. The expression of individual genes was normalized to the relative expression of trout housekeeping gene elongation factor 1α (EF-1α), and the expression levels were calculated using the 2^-ΔCt^ method, where ΔCt is determined by subtracting the EF-1α value from the target Ct. No template negative controls and *minus* reverse transcriptase controls were included in all the experiments.

### Statistical analysis

Statistical analyses were performed using the Graphpad prism version 6 (Graphpad software). All values were verified to be normally distributed. In all experiments, one-way ANOVA followed by Tukey’s test as a post-hoc was performed. The differences between the mean values were considered significant on different degrees, where * means *P* ≤ 0.05, ** means *P* ≤ 0.01 and *** means *P* ≤ 0.005.

## Supplementary information

Supplementary Information.
