# Supplementary material for: Aeromonas salmonicida activates rainbow trout IgM+ B cells signalling through Toll like receptors
Source: Sci Rep. 2020 Oct 8;10:16810. doi: 10.1038/s41598-020-73999-w (PMC7545209; doi:10.1038/s41598-020-73999-w)
Supplement: Supplementary file 1 — Supplementary Information. [file 41598_2020_73999_MOESM1_ESM.pptx]

## Slide 1
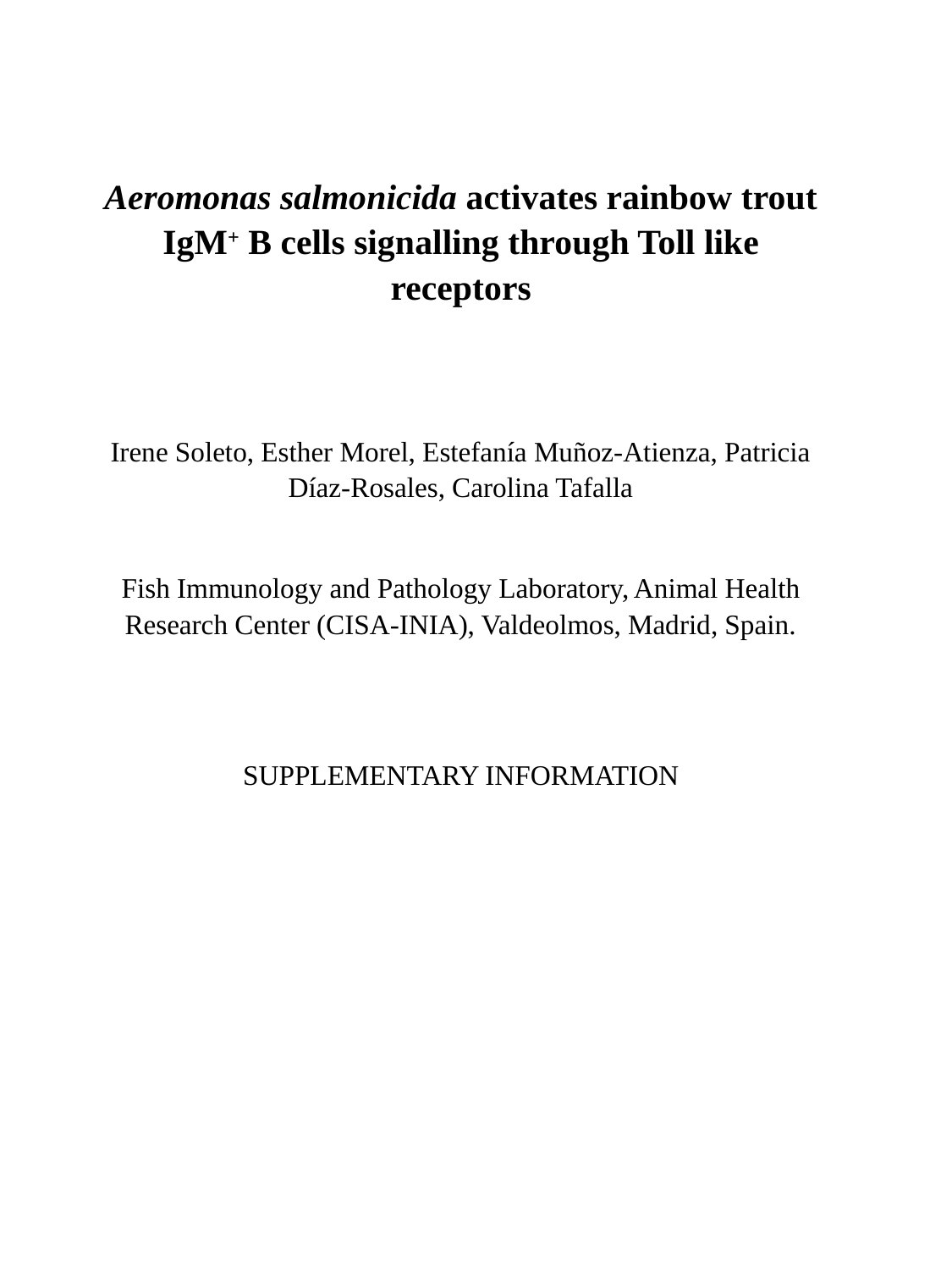

Aeromonas salmonicida activates rainbow trout IgM+ B cells signalling through Toll like receptors
Irene Soleto, Esther Morel, Estefanía Muñoz-Atienza, Patricia Díaz-Rosales, Carolina Tafalla
Fish Immunology and Pathology Laboratory, Animal Health Research Center (CISA-INIA), Valdeolmos, Madrid, Spain.
SUPPLEMENTARY INFORMATION

## Slide 2
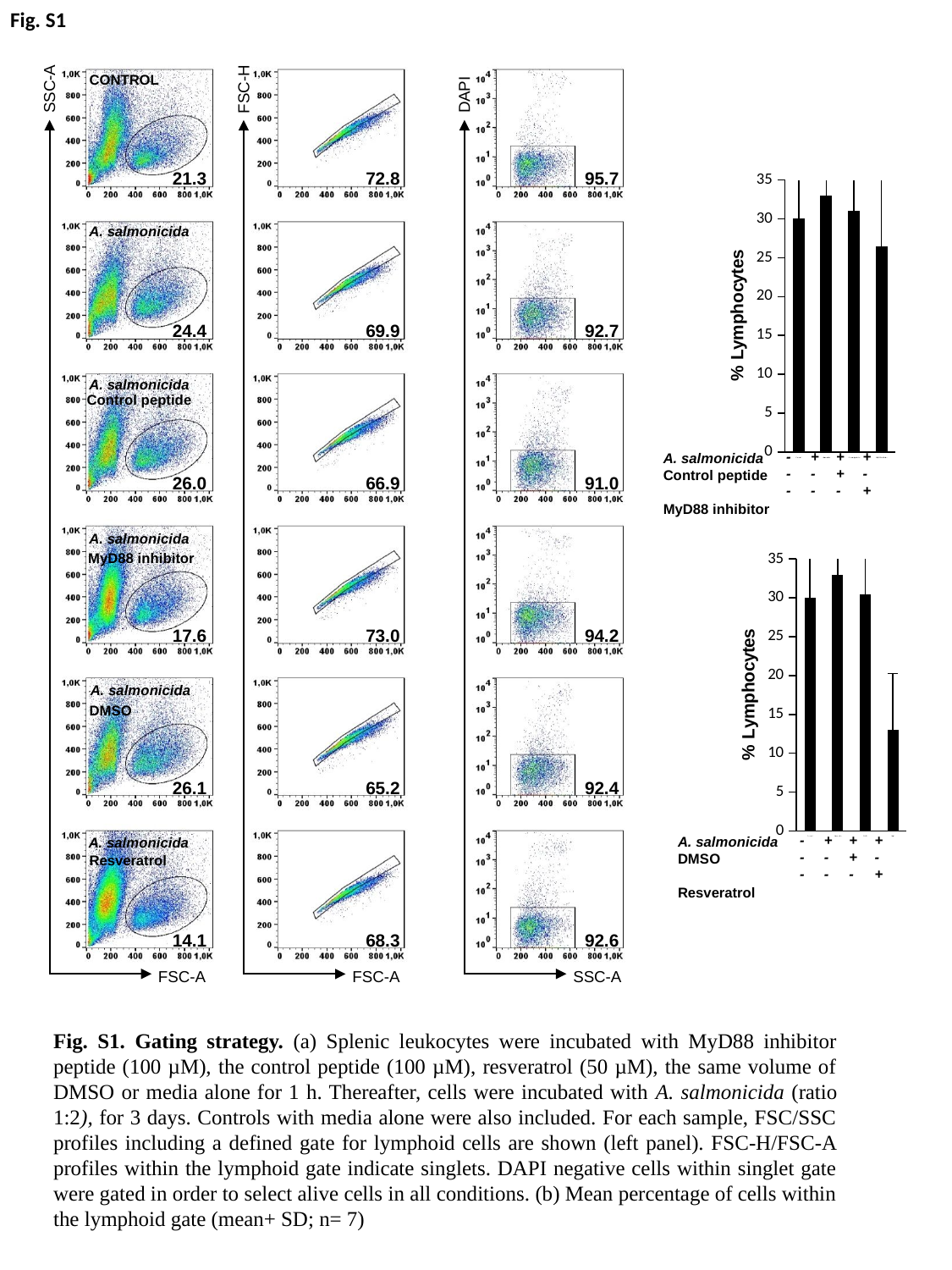

Fig. S1
SSC-A
FSC-A
DAPI
SSC-A
FSC-H
FSC-A
CONTROL
21.3
72.8
95.7
### Chart
| Category | |
|---|---|
| Control | 29.972857142857144 |
| A. salmonicida | 32.97857142857143 |
| Control peptide | 30.978571428571428 |
| MyD88 inhibitor | 26.39428571428571 |-
-
-
+
-
-
+
+
-
+
-
+
A. salmonicida
Control peptide
MyD88 inhibitor
A. salmonicida
24.4
69.9
92.7
A. salmonicida
Control peptide
26.0
66.9
91.0
A. salmonicida
MyD88 inhibitor
### Chart
| Category | |
|---|---|
| Control | 29.972857142857144 |
| A. salmonicida | 32.97857142857143 |
| DMSO | 30.40142857142857 |
| Resveratol | 12.974285714285713 |-
-
-
+
-
-
+
+
-
+
-
+
A. salmonicida
DMSO
Resveratrol
17.6
73.0
94.2
A. salmonicida
DMSO
26.1
65.2
92.4
A. salmonicida
Resveratrol
14.1
68.3
92.6
Fig. S1. Gating strategy. (a) Splenic leukocytes were incubated with MyD88 inhibitor peptide (100 µM), the control peptide (100 µM), resveratrol (50 µM), the same volume of DMSO or media alone for 1 h. Thereafter, cells were incubated with A. salmonicida (ratio 1:2), for 3 days. Controls with media alone were also included. For each sample, FSC/SSC profiles including a defined gate for lymphoid cells are shown (left panel). FSC-H/FSC-A profiles within the lymphoid gate indicate singlets. DAPI negative cells within singlet gate were gated in order to select alive cells in all conditions. (b) Mean percentage of cells within the lymphoid gate (mean+ SD; n= 7)

## Slide 3
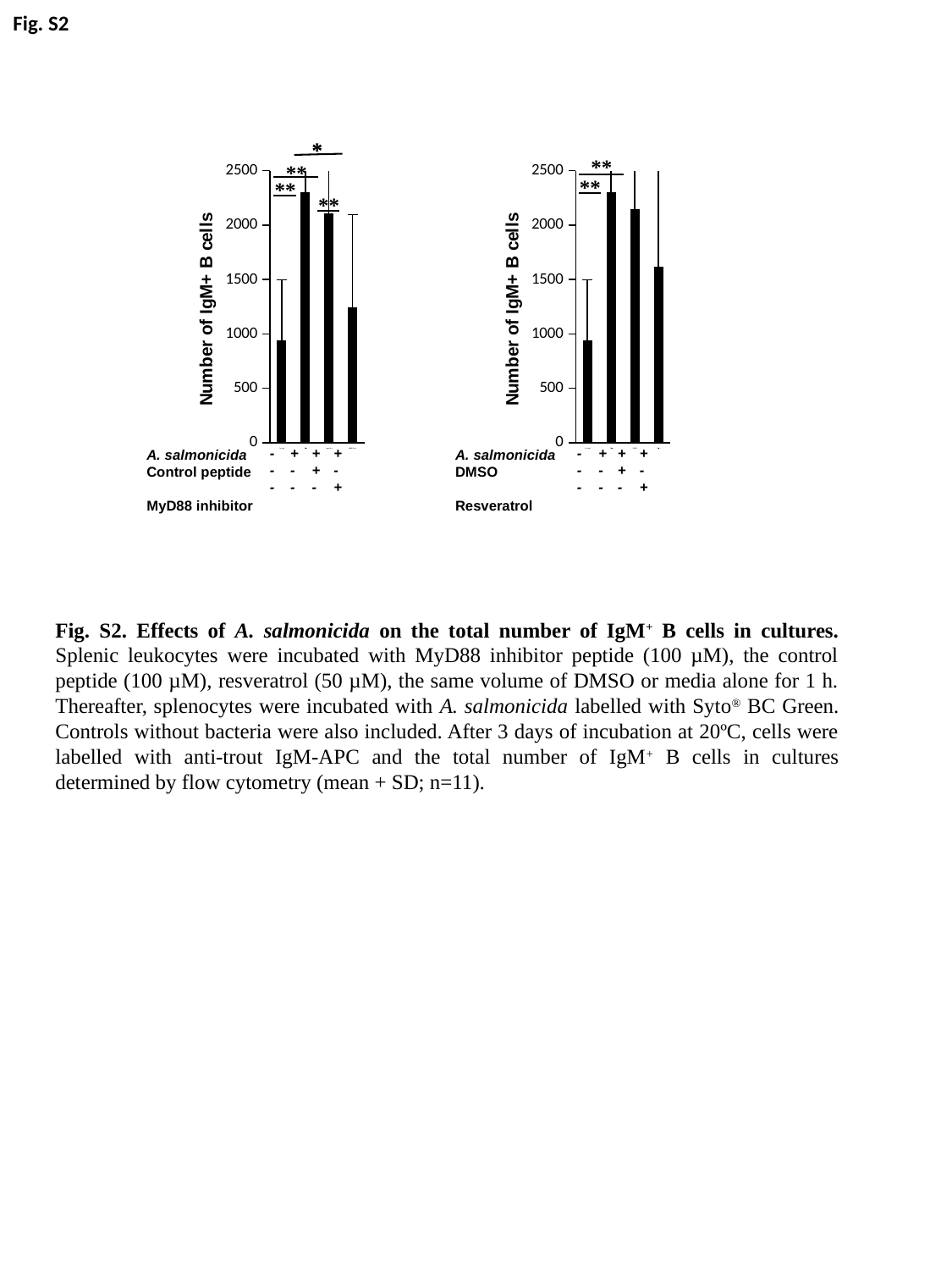

Fig. S2
*
*
**
**
### Chart
| Category | |
|---|---|
| Control | 940.8 |
| Bacteria | 2306.8 |
| peptide | 2107.25 |
| MyD88 inh | 1245.8333333333333 |
### Chart
| Category | |
|---|---|
| Control | 940.8 |
| Bacteria | 2306.8 |
| DMSO | 2147.0 |
| Resveratol | 1621.4166666666667 |**
**
**
-
-
-
+
-
-
+
+
-
+
-
+
A. salmonicida
Control peptide
MyD88 inhibitor
-
-
-
+
-
-
+
+
-
+
-
+
A. salmonicida
DMSO
Resveratrol
Fig. S2. Effects of A. salmonicida on the total number of IgM+ B cells in cultures. Splenic leukocytes were incubated with MyD88 inhibitor peptide (100 µM), the control peptide (100 µM), resveratrol (50 µM), the same volume of DMSO or media alone for 1 h. Thereafter, splenocytes were incubated with A. salmonicida labelled with Syto® BC Green. Controls without bacteria were also included. After 3 days of incubation at 20ºC, cells were labelled with anti-trout IgM-APC and the total number of IgM+ B cells in cultures determined by flow cytometry (mean + SD; n=11).

## Slide 4
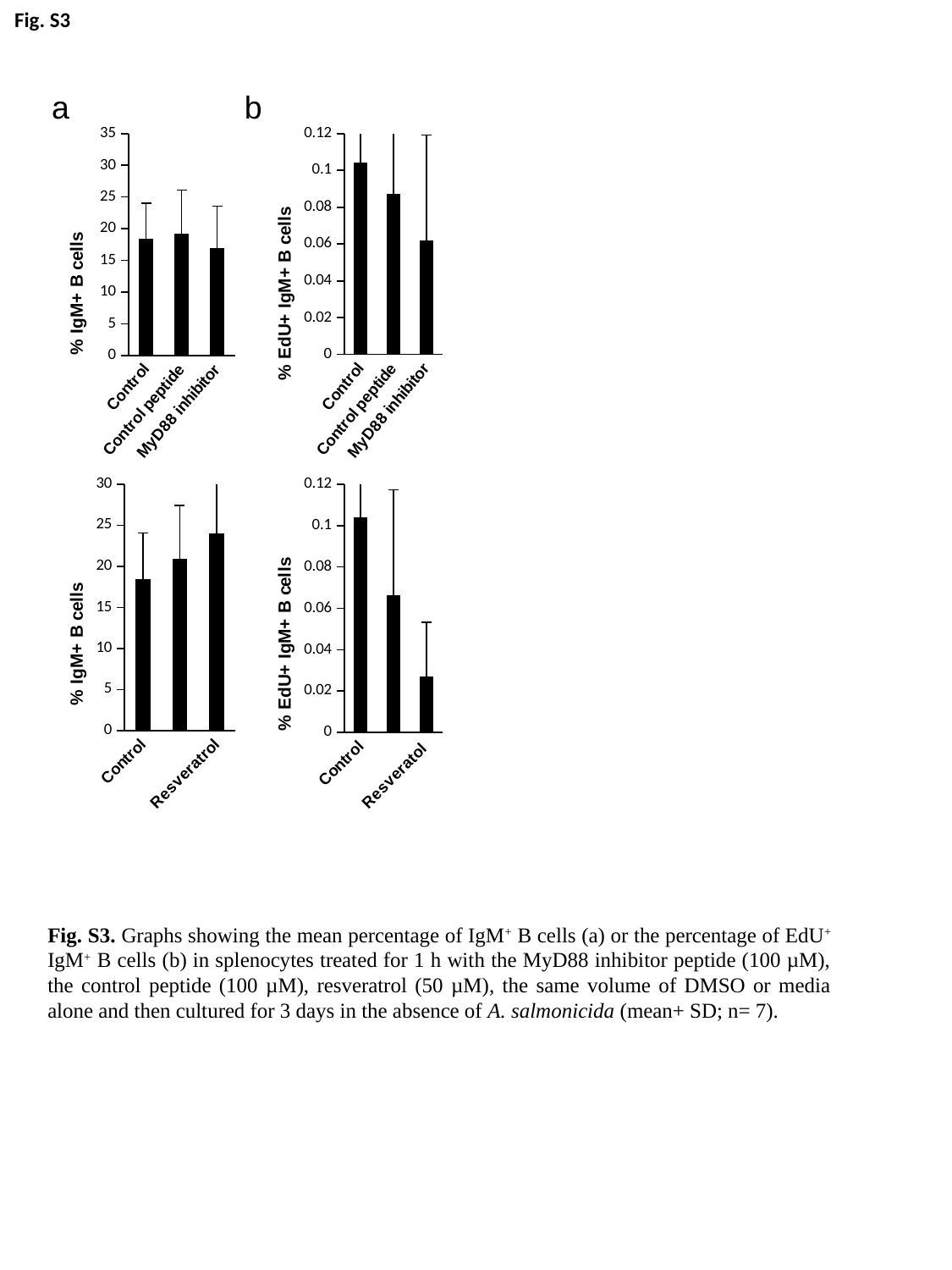

Fig. S3
a
b
### Chart
| Category | |
|---|---|
| Control | 18.442857142857147 |
| Control peptide | 19.25714285714286 |
| MyD88 inhibitor | 17.0 |
### Chart
| Category | |
|---|---|
| Control | 0.10416666666666664 |
| Control peptide | 0.08733333333333332 |
| MyD88 inhibitor | 0.06183333333333333 |
### Chart
| Category | |
|---|---|
| Control | 18.442857142857147 |
| DMSO | 20.87142857142857 |
| Resveratrol | 24.028571428571432 |
### Chart
| Category | |
|---|---|
| Control | 0.10416666666666664 |
| DMSO | 0.06633333333333334 |
| Resveratol | 0.02683333333333333 |Fig. S3. Graphs showing the mean percentage of IgM+ B cells (a) or the percentage of EdU+ IgM+ B cells (b) in splenocytes treated for 1 h with the MyD88 inhibitor peptide (100 µM), the control peptide (100 µM), resveratrol (50 µM), the same volume of DMSO or media alone and then cultured for 3 days in the absence of A. salmonicida (mean+ SD; n= 7).

## Slide 5
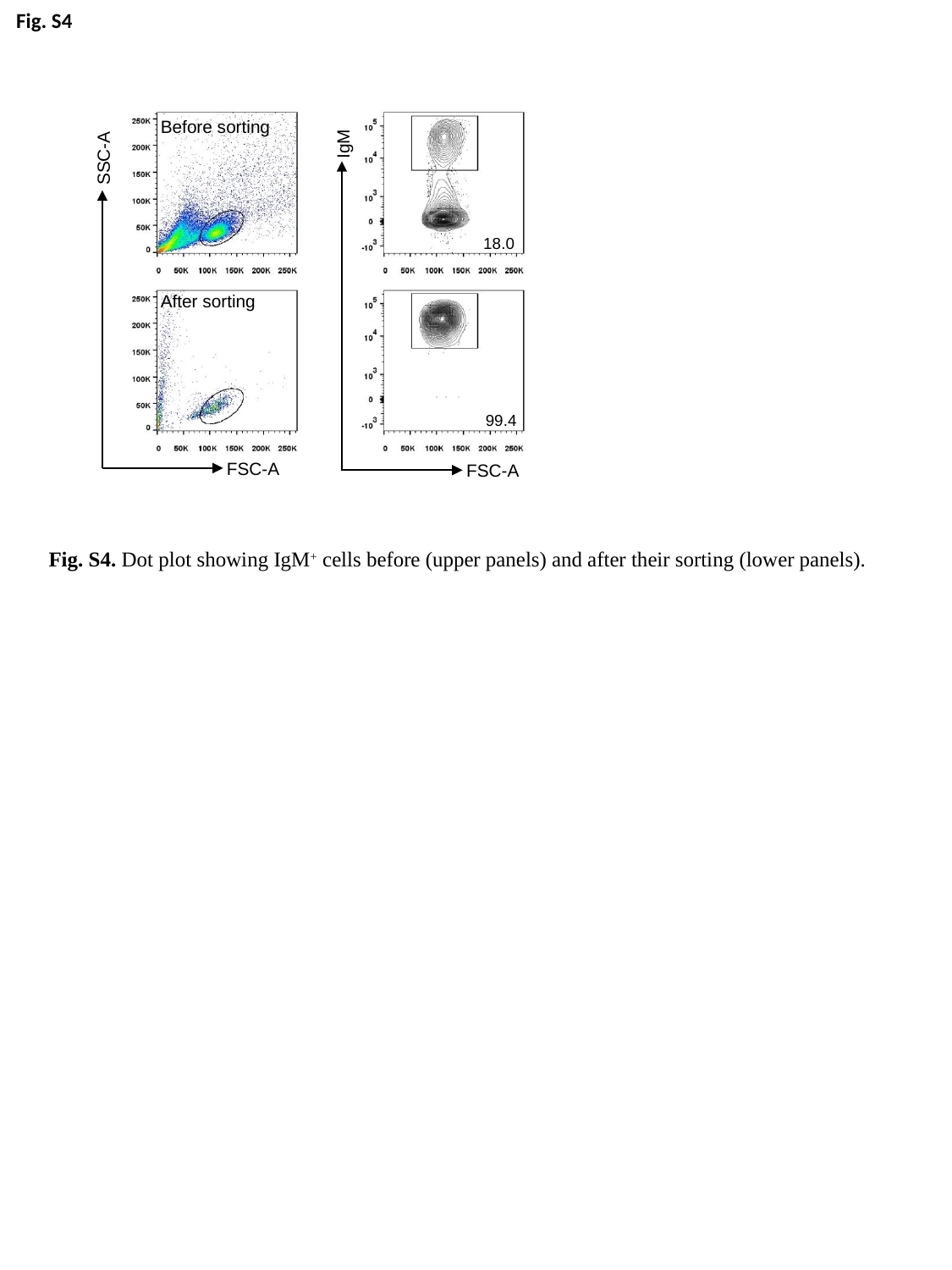

IgM
FSC-A
SSC-A
FSC-A
Fig. S4
Before sorting
18.0
After sorting
99.4
Fig. S4. Dot plot showing IgM+ cells before (upper panels) and after their sorting (lower panels).
